# Risk factors for prolonged air leak and need for intervention following lung resection

**DOI:** 10.1093/icvts/ivab243

**Published:** 2021-09-18

**Authors:** Aaron R Dezube, Daniel P Dolan, Emanuele Mazzola, Suden Kucukak, Luis E De Leon, Raphael Bueno, M Blair Marshall, Michael T Jaklitsch, Matthew M Rochefort

**Affiliations:** Division of Thoracic and Cardiac Surgery, Brigham and Women’s Hospital, Boston, MA, USA; Division of Thoracic and Cardiac Surgery, Brigham and Women’s Hospital, Boston, MA, USA; Department of Data Sciences, Dana Farber Cancer Institute, Boston, MA, USA; Division of Thoracic and Cardiac Surgery, Brigham and Women’s Hospital, Boston, MA, USA; Division of Thoracic and Cardiac Surgery, Brigham and Women’s Hospital, Boston, MA, USA; Division of Thoracic and Cardiac Surgery, Brigham and Women’s Hospital, Boston, MA, USA; Division of Thoracic and Cardiac Surgery, Brigham and Women’s Hospital, Boston, MA, USA; Division of Thoracic and Cardiac Surgery, Brigham and Women’s Hospital, Boston, MA, USA; Division of Thoracic and Cardiac Surgery, Brigham and Women’s Hospital, Boston, MA, USA

**Keywords:** Air leak, Thoracic surgery, Intervention, Chest tube

## Abstract

**OBJECTIVES:**

Prolonged air leak (PAL; >5 days) following lung resection is associated with postoperative morbidity. We investigated factors associated with PAL and PAL requiring intervention.

**METHODS:**

Retrospective review of all patients undergoing lobectomy, segmentectomy or wedge resection from 2016 to 2019 at our institution. Bronchoplastic reconstructions and lung-volume reduction surgeries were excluded. Incidence and risk factors for PAL and PAL requiring intervention were evaluated.

**RESULTS:**

In total, 2384 patients were included. PAL incidence was 5.4% (129/2384); 22.5% (29/129) required intervention. PAL patients were more commonly male (56.6% vs 39.7%), older (mean age 69 vs 65 years) and underwent lobectomy or thoracotomy (all *P* < 0.001). Patients with PAL had longer length of stay (9 vs 3 days), more discharge needs and increased odds of complication (all *P* < 0.050).

Twenty-nine patients required intervention (9 chest tubes; 4 percutaneous drains; 16 operations). In 50% of operative interventions, an air leak source was identified; however, the median time from intervention to resolution was 13 days. Patients requiring intervention had increased steroid use, lower diffusion capacity for carbon monoxide and twice the length of stay versus PAL patients (all *P* < 0.050).

On univariable analysis, forced expiratory volume in 1 s (FEV1) <40%, diffusion capacity for carbon monoxide <50%, steroid use and albumin <3 had increased odds of intervention (*P* < 0.050).

**CONCLUSIONS:**

Age, gender and operative technique were related to PAL development. Patients with worse forced expiratory volume in 1 s or diffusion capacity for carbon monoxide, steroid use or poor nutrition were less likely to heal on their own, indicating a population that could benefit from earlier intervention.

**Figure:**
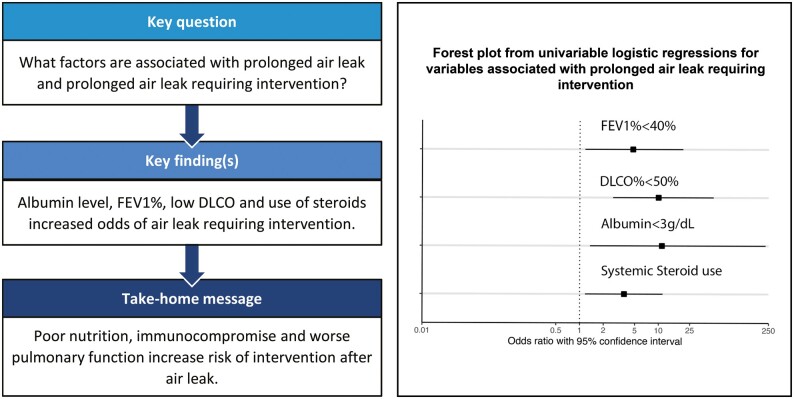

## INTRODUCTION

Postoperative air leak after lung resection is one of the most common problems that thoracic surgeons manage. Approximately half of lung resection patients experience air leak immediately after surgery which decreases to 5–20% by postoperative Day 5, excluding those patients who undergo lung-volume reduction surgery [1–4]. Air leaks that persist for longer than 5 days are tracked in the Society of Thoracic Surgery Database and this 5-day duration is commonly regarded as the definition for a prolonged air leak (PAL) after thoracic surgery [5]. PALs are a burden to the healthcare system secondary to increasing inpatient length of stay (LOS) and the associated cost of that LOS and various required interventions [6,7]. While the development of unidirectional dry seal drainage systems such as the Heimlich valve, Atrium Pneumostat™ and Atrium Express Mini™ has been instrumental in decreasing LOS, by allowing patients with small persistent air leaks to be discharged from the hospital and managed on an outpatient basis, these patients still require visiting nursing assistance and frequent use of healthcare resources until the PAL is resolved [8,9].

Several studies have identified risk factors for PAL [7,10–12]. These include lower body mass index, presence of pleural adhesions, surgeon experience and higher early postoperative air leak flow (ml/min) as measured on a digital chest drainage system [10,12]. However, in practice, previous PAL scores based on preoperative factors remain limited, with high risk of false positives and low-positive predictive value [13].

The incidence of severe PAL, such that they require an intervention in order to heal, has been noted to be ∼5% after pulmonary resection [14]. However, literature on this subset of patients is scarce, despite requiring greater healthcare resources and having increased morbidity and mortality related to the secondary interventions. Given the paucity of data regarding these challenging PALs, the objective of this study was to identify risk factors for PAL in a large, single institution, consecutive patient series and to further identify the risk factors for those patients who required advanced interventions for PAL treatment.

## METHODS

Institutional Review Board (#2014P002478) approval was obtained from our institution. Informed consent was waived by our Institutional Review Board. We retrospectively reviewed all patients, from our prospectively maintained Division of Thoracic Surgery morbidity and mortality database who underwent a lobectomy, segmentectomy or wedge resection from May 2016 through December 2019. We excluded all cases where lung resections were not the primary procedure, and those who underwent bronchial sleeve lobectomy, lung-volume reduction surgery, bi-lobectomy or had bronchoplastic closure.

Details of our perioperative management are in Supplementary Data S1. All patients in our study underwent either an open, video-assisted thoracoscopic surgery (VATS) or robotic-assisted thoracoscopic surgery (RATS). Use of Progel Pleural Air Leak Sealant (CR Bard, Warwick, RI, USA), pleural tent, or muscle flap buttress was performed for staple line reinforcement at the discretion of the attending surgeon.

### Postoperative care

Patients were extubated and transferred to either the intensive care unit (ICU) or the Thoracic Intermediate Care Unit with a chest tube and or Blake™ drain set to −10 to −40 mmHg suction based on surgeon preference. Patients were followed with serial chest radiography. Based on air leak, chest drain output (<250–300 ml/24 h) and chest radiography findings, chest tubes and silicone drains were removed when appropriate. Post-pull chest radiography was routinely performed after removal of the last chest drain. PAL was noted and defined as an air leak lasting greater than 5 days. Patients with asymptomatic PALs were frequently discharged with an Atrium Pneumostat™ Chest Drain Valve and, once the air leak resolved, their tube was removed on an outpatient basis.

### Variables and outcomes

We considered demographics (*age, gender*), operative details (*lobectomy, segmentectomy, wedge resection*), method of resection (*open, VATS, or RATS*), postoperative complications (overall and by Clavien–Dindo grade II–IV excluding air leak as a complication), hospital LOS, and discharge disposition (*home, home with services, skilled nursing facility, rehab/extended care* *and other*).

In patients who developed a PAL, additional variables were collected when available: preoperative pulmonary function tests (*FEV1%, FVC%, DLCO%*), albumin, smoking status (*non-smoker* *or current smoker or within 30 days of surgery*), systemic steroid use, neoadjuvant radiation to ipsilateral side of surgery, neoadjuvant chemotherapy and history of prior cardiothoracic surgery on same side. Additional operative details that were collected included: lysis of adhesions, whether the procedure was a reoperation on same side, presence of air leak at the time of surgery and if an adjunct air leak method was utilized (*gel, muscle flap*). Lastly, the additional postoperative variables collected included: duration of PAL, indication for reintervention for PAL, time from index procedure to reintervention, findings at reintervention and procedure performed (*chest tube, percutaneous drainage or operative procedure and details*), duration from intervention to air leak resolution, days to discharge from intervention, if PAL was the primary cause of delay of discharge, and if an Atrium Pneuomostat™ was required on discharge.

Details of our complication classifications and quality assurance protocols are supplied as Supplementary Data S2. Patients with missing observations in any of the considered variables were excluded from the analysis. Multiple imputation was considered for variables with a high rate of missing observation [i.e. diffusion capacity for carbon monoxide (DLCO)], but missing data were not random therefore the decision was made not to perform this, as it would lead to bias observations

### Statistical analysis

Patients were divided into those with a PAL and those without and we compared the 2 groups. Further analysis was performed on those who required intervention for PAL and those who did not. Wilcoxon rank-sum test was used for the comparison of continuous variables, while chi-squared test (or Fisher’s exact test when variables were = *n* < 10) were used to compare categorical variables.

Univariable and multivariable logistic regression models were estimated to assess the odds of (i) development of overall complication (excluding PAL) for the overall cohort and the subset of patients with PAL, (ii) PAL and separately (iii) of the need for intervention in those patients who developed PAL. The selection criterion of the variables included in the initial univariable model was based on a priori clinical relevance. Inclusion into the multivariable model was based on statistical significance (*P* < 0.05) in the univariable model; variables that led to unstable estimates of the odds ratios (ORs) due to low event rate or possible unobserved correlation were removed from the models. Statistical significance was considered with a *P*-value <0.05. All analysis was performed using Stata 16 (College Station, TX, USA: StataCorp LLC) [15] and R 4.0.3 statistical software (R Core Team 2020).

## RESULTS

In total, 2384 patients were analyzed. The cohort had a median age of 67 years [interquartile range (IQR) 59–74] and was primarily female (59.4%). Procedures were 777 lobectomies (32.6%), 278 segmentectomies (11.7%), and 1329 wedge resections (55.8%). Surgical technique was primarily VATS (84.2%), followed by thoracotomy (11.8%), then RATS (4%). Median LOS was 3 days (IQR 2) with 94% discharged home or home with services. The overall complication rate was 17.6% with a PAL rate of 5.4% (*n* = 129), of which 22.5% (*n* = 29/129) required intervention. PAL rates were higher in lobectomies [9.9% (*n* = 77/777) vs 6.1% (*n* = 17/278) vs 2.6% (*n* = 35/1329) for lobectomy, segmentectomy and wedge resection, respectively; *P* < 0.001] and thoracotomies [12.1% (*n* = 34/281) vs 4.6% (*n* = 92/2008) vs 3.2% (*n* = 3/95) for thoracotomy, VATS, and RATS, respectively; *P* < 0.001].

Patients with PAL had longer hospital LOS [median 9 (IQR 7–14) vs 3 days (IQR 2–4)], and higher rates of discharge to inpatient rehabilitation or discharge home with services, as well as increased Clavien–Dindo overall and grade II–V complications (*P* < 0.001 for overall and grade II–IV complications and *P* = 0.016 for grade V complication, respectively) (Table 1). On the whole dataset of 2384 patients, in both univariable and multivariable logistic regressions, PAL was estimated to be associated with increased odds of overall complication [OR 4.59 (95% confidence interval (CI): 3.19–6.61) and 3.26 (95% CI: 2.21–4.80), respectively; both *P* < 0.001; Supplementary Material, Table S1].

**Table 1:** Baseline demographics, operative details and complications of patients undergoing resection with or without prolonged air leak

	Prolonged air leak (*n* = 129)	No prolonged air leak (*n* = 2255)	*P*-value
LOS in days, median (IQR)	9 (7–14)	3 (2–4)	<0.001
Discharge disposition, *n* (%)			<0.001
Home	21 (16.5)	1104 (49.0)	
Home w/services	92 (72.4)	135 (46.0)	
Skilled nursing facility	4 (3.2)	15 (0.7)	
Rehab	9 (7.1)	92 (4.1)	
Other	1 (0.8)	6 (0.3)	
Complications, *n* (%)^a^			
Overall	60 (46.5)	359 (15.9)	<0.001
Grade II	47 (36.4)	296 (13.1)	<0.001
Grade III	22 (17.1)	93 (4.1)	<0.001
Grade IV	9 (7.0)	41 (1.8)	<0.001
Grade V	2 (1.6)	2 (0.1)	0.016

a Excluded air leak.

IQR: interquartile range; LOS: length of stay.

Univariable logistic regression models examining the development of PAL only (Table 2) showed higher odds ratio for PAL development in patients 70 years old or greater (ref < 70) and undergoing segmentectomy or lobectomy (ref. wedge resection). Factors associated with lower rates of PAL included female gender (ref. male), and minimally invasive techniques including VATS or RATS (ref. thoracotomy). In the corresponding multivariable model, segmentectomy (OR 2.41; 95% CI 1.32–4.39) and lobectomy (OR 3.76; 95% CI 2.42–5.84; ref wedge) remained associated with increased odds of PAL development, while female gender (ref male; OR 0.51; 95% CI 0.35–0.72), and RATS (ref thoracotomy; OR 0.24; 95% CI 0.07–0.82) were associated with lower odds of PAL development (Table 2).

**Table 2:** Univariable and multivariable logistic regression of prolonged air leak development

	Univariable			Multivariable		
Variable name	Odds ratio	95% confidence interval	*P*-value	Odds ratio	95% confidence interval	*P*-value
Age 70 + (ref <70)	1.44	1.01–2.06	0.045	1.38	0.96–1.99	0.079
Female gender (ref male)	0.51	0.35–0.72	<0.001	0.50	0.35–0.72	<0.001
Resection type						
Segmentectomy (ref. wedge)	2.41	1.33–4.36	0.004	2.41	1.32–4.39	0.004
Lobectomy (ref wedge)	4.07	2.70–6.13	<0.001	3.76	2.42–5.84	<0.001
Method						
Thoracoscopic (ref open)	0.35	0.23–0.53	<0.001	0.64	0.41–1.01	0.055
Robotic (ref. open)	0.24	0.07–0.79	0.019	0.24	0.07–0.82	0.022

### Prolonged air leak requiring intervention (reduced dataset, *n* = 129 observations)

Twenty-nine patients required intervention for their PAL, which consisted of 9 chest tubes (31%), 4 percutaneous drains (13.8%) and 16 reoperations (55.2%). Of those patients who ultimately required intervention, 89.7% had no significant air leak at the end of their initial procedure. In the 3 cases that did have a concern for an air leak at the end of their initial procedure, reinforcement procedures done intraoperatively included placement of a pericardial fat pad, intercostal muscle flap or pleural tent, respectively.

The median time from index procedure to reintervention was 7.5 days (IQR 6–11; Table 3). Reasons for intervention included PAL without clinical improvement or PAL with worsening subcutaneous emphysema (48.3%), symptomatic pneumothorax (31.0%) or other (20.7%) including lobar collapse, empyema, chylothorax, dyspnoea or increase oxygen requirement. In 50% of operative interventions a presumed air leak source was identified, which was managed, respectively, with VATS blebectomy or wedge resection in 2 cases, thoracotomy and pectoralis flap for a broncho-pleural fistula, open pleural tent, Progel™ placement or DuraSeal™, additional chest tube placement in 3 cases, and 1 case of endobronchial valve placement with the creation of a Clagett window (open window thoracostomy in the lateral aspect of the chest for empyema management).

**Table 3:** Outcomes of patients after lung resection with air leak in those with or without need for intervention

Variable name	PAL requiring intervention	PAL not requiring intervention	*P*-value
	*n* = 29	*n* = 100	
Outcomes			
Intervention, *n* (%) Bedside chest tube Pigtail by interventional radiology Operative intervention	9 (31.0%)	N/A	N/A
	4 (13.8%)		
	16 (55.2		
Time from index to intervention in days, median (IQR)	7.5 (6–11)	N/A	N/A
Reason for intervention Air leak Symptomatic pneumothorax Other	14 (48.3%) 9 (31.0%) 6 (20.7%)	N/A	N/A
Duration of PAL in days, median (IQR)	18.5 (14–28.5)	10 (6–16.8)	<0.001
Duration of PAL from intervention in days, median (IQR)	14 (5–20.5)	N/A	N/A
Delay of discharge due to PAL	17 (58.6%)	92 (92.0%)	<0.001
Discharged with pneumostat, *n* (%)	15 (51.7%)	39 (39.0%)	0.447
LOS, days, median (IQR)	16 (12–22)	8 (7–11)	<0.001
Days to discharge from intervention, median (IQR)	8.5 (5–15)	N/A	N/A
Discharge disposition, *n* (%)			
Home	5 (17.2%)	16 (16.3%)	
Home w/service	15 (51.7%)	77 (78.6%)	<0.001
Skilled nursing facility	2 (6.9%)	2 (2.0%)	
Extended care/rehab	6 (20.7%)	3 (3.1%)	
Other	1 (3.5%)	0 (0.0%)	
Complication rate, *n* (%)^a^			
Overall^b^	21 (72.4%)	39 (39.0%)	<0.001
Grade II^b^	13 (44.8%)	34 (34.0%)	0.381
Grade III^b^	14 (48.3%)	8 (8.0%)	<0.001
Grade IV	4 (13.8%)	5 (5.0%)	0.114
Grade V	0 (0%)	2 (2.0%)	>0.999

a Clavien-Dindo Classification.^22^

b Excluded air leak.

LOS: length of stay; PAL: prolonged air leak; SD: standard deviation.

Median PAL duration was 18.5 (IQR 14–28.5) days in those requiring intervention versus 10 (IQR 8–16.75) days in those without intervention (*P* < 0.001). Median time from intervention to resolution of air leak was 14 days (IQR 5–20.5); however, symptom resolution occurred in all after intervention. The median hospital LOS was 16 (IQR 12–22) days in those requiring intervention versus 8 (IQR 7–11) days in the non-intervention cohort (*P* < 0.001). However, delay in discharge was attributed solely to air leak in only 58.6% of cases requiring intervention while the primary reason (92%) for discharge delay was air leak in the non-intervention cohort (*P* < 0.001). The median time from intervention to discharge was 8.5 days (IQR 5–15).

PAL intervention patients, as compared to PAL patients without intervention had higher overall complications excluding air leak (72.4% vs 39.0%; *P* < 0.001), but this was only statistically significant for grade III complications (48.3% vs 8%; *P* < 0.001). In a univariable logistic regression model, PAL intervention (Ref PAL not requiring intervention) was associated with increased odds of complication (OR 4.11; 95% CI 1.71–10.72; *P* = 0.002), but once adjusted for DLCO in our multivariable model this was no longer significant (*P* = 0.056; Supplementary Material, Table S2). The estimates are quite unstable possibly due to the presence of only 11.6% patients (*n* = 8/69) who got PAL intervention with no complications.

On discharge, patients undergoing intervention still required a Pneumostat™ in 51.7% of cases. PAL patients who did not require intervention went home with a Pneumostat™ in 39.0% of cases (*P* = 0.447). Patients requiring intervention had significantly higher rates of discharge to skilled nursing facility (6.9% vs 2.0%), extended care or rehab (20.7% vs 3.1%) and lower rates of discharge home with services (51.7% vs 78.6%; *P* < 0.001).

Univariable logistic regression to assess the odds of PAL requiring intervention compared to PAL without intervention is somewhat challenging secondary to the relatively low event rate. Despite this, we found increased odds of preoperative FEV1% <40% (ref >40%; OR 4.74; 95% CI 1.18–19.02, *P* = 0.028), DLCO <50% (ref >50%; OR 10; 95% CI 2.39–41.84, *P* = 0.002), steroid use (OR 3.66; 95% CI 1.199–11.169; *P* = 0.023) and albumin <3 (ref >3; OR 11.04; 95% CI 1.100–110.7; *P* = 0.041; Table 4). The already wide CIs in the univariable models were possibly related, as mentioned, to low event rates in this subset of patients: this prevented us from proceeding with a multivariable model as it would generate unstable results, the interpretation of which would be challenging and unreliable.

**Table 4:** Univariable logistic regression of prolonged air leak requiring intervention

Variable	Odds ratio	95% confidence interval	*P*-value
Age <70 years old (reference ≥70 years old)	0.45	0.19–1.06	0.068
Gender, female (reference male)	1.08	0.47–2.48	0.861
FEV1% <40% (reference ≥40%)	4.74	1.18–2.48	0.028
FVC equal of greater 110% (reference <110%)	0.56	0.07–4.79	0.594
DLCO% <50% (reference ≥50%)	10	2.49–41.84	0.002
Albumin <3 g/dl (reference ≥3 g/dl)	11.04	1.10–110.7	0.041
Current smoker (reference quit >30 days prior)	0.68	0.21–2.19	0.521
Systemic steroid use, yes (reference no)	3.66	1.20–11.17	0.023
Ipsilateral radiation therapy, yes (reference no)	1.30	0.38–4.42	0.680
Neoadjuvant Chemotherapy, yes (reference no)	1.75	0.60–5.09	0.308
Prior ipsilateral Cardiothoracic surgery, yes (reference no)	0.51	0.18–1.47	0.211
Degree of resection (reference wedge)			
Segmentectomy	0.54	0.13–2.28	0.398
Lobectomy	0.66	0.26–1.64	0.367
Method (ref thoracotomy)			
Thoracoscopic	1.56	0.57–4.23	0.387
Robotic			
Lysis of adhesions or re-do, yes (ref no)	0.81	0.35–1.85	0.615

DLCO: diffusion capacity for carbon monoxide; FEV1: forced expiratory volume in 1 s; FVC: forced vital capacity.

## DISCUSSION

PAL is a common postoperative thoracic surgical complication with a significant healthcare burden. Our study investigated the incidence and risk factors for the development of (PAL) following lung resection for common thoracic surgery operations. We found that PAL patients incurred three times longer hospital LOS compared to those who did not develop a PAL, had higher complication rates apart from air leak itself, and required more outpatient services. In our work procedure type, and method of resection were correlated with air leak development. Compared to thoracotomies, PAL was less common after VATS resections and least common after RATS resections.

The incidence of PAL in our study was 5.7% which is somewhat lower than 10–25% quoted in the literature [14,16]. We noted a similar rate of severe PAL in our patients, defined as the subset of PAL patients that required an intervention, 22.5% vs 26%; however, this subset only comprised 1.2% of all patients in our cohort vs 4.8% of the cohort as reported by Liang’s group [14]. Our LOS was shorter for those patients who did not require intervention, but similar for the PAL patients requiring intervention, with LOS in the 11- to 30-day range. Interventions did not appear to lead to immediate air leak resolution and earlier discharge in either study. We found an increased risk of PAL associated with open surgery in contrast to the work by Liang, but similar to the findings of Attaar *et al.* [10,14]. Furthermore, our risk of PAL in lobectomy patients is similar to prior studies [10–12,14]. It is encouraging that the incidence of PAL in our cohort was lower while keeping a similar rate of those patients requiring intervention.

Surgery for PAL was helpful in symptomatic treatment, but surgical exploration did not lead to immediate resolution of air leak. Multiple methods have been studied for both prophylaxis against PAL as well for their management after they occur; however, there is no clear direction for timing and indications for intervention for PAL after surgery [17–20].

Preoperative exercise-based intervention, ‘prehabilitation’, has been discussed as a possible way of optimizing patients prior to major cardiothoracic and abdominal surgery [21]. In our subanalysis of PAL patients who required intervention, we identified worse FEV1%, worse DLCO%, lower albumin and increased steroid use as factors with increased odds of requiring intervention. All these factors are potential targets for prehabilitation intervention. However, given the time-sensitive nature of the diagnosis, staging and ultimate surgical management of lung cancer, which constitutes the vast majority of indications for lung resection, the ability to prehabilitate these patients is significantly limited. Therefore, the identification of high-risk features can assist in determining those patients that may benefit from earlier intervention. Alternatively, this can identify those patients at low risk for symptom development, who may safely be discharged with conservative outpatient management. In contrast to prior work, in our cohort, we found that having had prior surgery on the same side, extensive lysis of adhesions, and performing a re-do operation did not result in more severe air leaks and did not increase the odds of requiring intervention [10]. Additional areas of study regarding PAL could include early and aggressive optimization of nutrition to decrease the incidence of severe air leaks requiring intervention and randomization to early intervention versus ongoing watchful waiting for patients with high-risk features (lower FEV1%, lower DLCO%, systemic steroid use) to determine the cohort that truly benefits from intervention.

### Limitations

Limitations of our study include low event rates harming the univariable models and preventing robust multivariable models for our interventional cohort. As this study was a single-centre retrospective, when to perform intervention and which intervention to perform were at the treating surgeon’s discretion as was performance of staple line reinforcement during the index procedure which may contribute to selection bias. We did not prospectively record the amount of negative pressure on drains, and this variable was not available for analysis. More data are needed to further refine risk factors for PAL and for those PAL that will require intervention with need for a prospective randomized study to determine the optimal treatment for PALs.

In conclusion, the incidence of PAL in our study appears to be lower than some more contemporary studies, which may reflect a greater proportion of cases being performed minimally invasively, but the incidence of those requiring intervention remains consistent indicating an area for further investigation. Demographics such as age and gender as well as operative technique were related to PAL development and PAL was associated with higher complications. Patients with worse FEV1, worse DLCO, steroid use and poor nutrition were less likely to heal on their own and more likely to develop symptoms, indicating a population that could benefit from earlier intervention.

## SUPPLEMENTARY MATERIAL

Supplementary material is available at *ICVTS* online.

## Funding

This study was supported in part by the generous donation of the Jack Mitchell Thoracic Oncology Fellowship.

**Conflict of interest:** All authors have no conflict of interest or financial disclosure with the exception of Raphael Bueno who has research grants with Medgenome, Roche, Verastem, Merck, Gristone, Epizyme, Siemens, Celsius, NCI, DoD, NIH and Patent/Equity in Navigation Sc.

### Author contributions

**Aaron R. Dezube:** Conceptualization; Data curation; Formal analysis; Funding acquisition; Investigation; Methodology; Project administration; Resources; Software; Supervision; Validation; Visualization; Writing—original draft; Writing—review & editing. **Daniel P. Dolan:** Conceptualization; Data curation; Formal analysis; Investigation; Methodology; Project administration; Resources; Software; Supervision; Validation; Visualization; Writing—original draft; Writing—review & editing. **Emanuele Mazzola:** Formal analysis; Methodology; Resources; Software; Validation; Writing—review & editing. **Suden Kucukak:** Conceptualization; Data curation; Formal analysis; Investigation; Methodology; Validation; Visualization; Writing—original draft; Writing—review & editing. **Luis E. De Leon:** Conceptualization; Data curation; Formal analysis; Investigation; Methodology; Resources; Validation; Visualization; Writing—original draft; Writing—review & editing. **Raphael Bueno:** Conceptualization; Formal analysis; Investigation; Methodology; Project administration; Resources; Validation; Visualization; Writing—original draft; Writing—review & editing. **M. Blair Marshall:** Conceptualization; Formal analysis; Investigation; Methodology; Supervision; Validation; Visualization; Writing—original draft; Writing—review & editing. **Michael T. Jaklitsch:** Conceptualization; Data curation; Formal analysis; Funding acquisition; Investigation; Methodology; Project administration; Resources; Supervision; Validation; Visualization; Writing—original draft; Writing—review & editing. **Matthew M. Rochefort:** Conceptualization; Data curation; Formal analysis; Funding acquisition; Investigation; Methodology; Project administration; Resources; Software; Supervision.

### Reviewer information

Interactive CardioVascular and Thoracic Surgery thanks Lucio Cagini, Po-Kuei Hsu, Francesco Zaraca and the other anonymous reviewers for their contribution to the peer review process of this article.

## Supplementary Material

ivab243_Supplementary_Data

## Notes

This abstract was presented as e-poster at the 57th annual STS meeting 29–31 January 2021.

## Abbreviations

**ABBREVIATIONS**
CI: Confidence interval
DLCO: Diffusing capacity for carbon monoxide
FEV1: Forced expiratory volume in 1 second
IQR: Interquartile range
LOS: Length of stay
OR: Odds ratio
PAL: Prolonged air leak
RATS: Robotic-assisted thoracoscopic surgery
VATS: Video-assisted thoracoscopic surgery
