# Supplementary material for: Risk factors for prolonged air leak and need for intervention following lung resection
Source: Interact Cardiovasc Thorac Surg. 2021 Sep 18;34(2):212–8. doi: 10.1093/icvts/ivab243 (PMC8766207; doi:10.1093/icvts/ivab243)
Supplement: ivab243_Supplementary_Data [file ivab243_supplementary_data.zip › 2dd Version Supplemental Data 1 and 2.docx]

**Supplemental appendix 1:** Perioperative management

Pre-operative work up included physical examination, hematologic and biochemical tests, electrocardiogram, chest X-rays (CXR), computed tomography (CT) scan of the chest, positron emission technology-CT scan (PET-CT), bronchoscopy, and endobronchial ultrasound (EBUS) or cervical mediastinoscopy when clinically appropriate. Pulmonary function tests (PFTs) were routinely obtained in all patients and forced expiration in 1 second (FEV1%), forced vital capacity (FVC%), FEV1/FVC, and diffusing capacity for carbon monoxide (DLCO%) values were evaluated and normalized according to age, gender, and height of the patient. Other testing was performed as indicated based on previously obtained results.

All patients were anesthetized by the same team of senior cardio-thoracic anesthesiologist and operated on under the supervision of the same team of experienced thoracic surgeons. Prophylactic antibiotic regimen was based on a standard pre-operative dose of 2000 mg of Cefazolin, that was repeated every 6 hours if surgery was still ongoing.

**Supplemental appendix 2:** Classification and quality assurance of complications

Postoperative outcomes and discharge dispositions were collected prospectively in a computerized database by the thoracic surgery team. Daily visits to the hospital wards by a trained data-manager were performed to assess postoperative morbidity and mortality, which included events within 30 days of surgery.  Morbidity was graded based on the Clavien-Dindo classification of surgical complications^22^. This system grades complications on a scale of 1-5. Grade I are minimal complications that require only minor intervention; interventions allowed in this grade include antiemetics, antipyretics, diuretics, and electrolytes. Grade II complications require pharmacological intervention not included in Grade I, or blood products. Grade III complications are those that require surgical, endoscopic or radiologic intervention; grade IV are those in which there is organ dysfunction, and/or requirement of ICU level of care; and finally Grade V are those that result in the patient’s death. Grade I-II complications are considered minor, while complications III-V are considered major events. Only complications Grade II or higher were recorded in this study. All complications had a standardized definition to ensure the same event was captured every time. There was no prospective recording of amount of negative suction pressure on chest drains.

All events were audited twice weekly, first by two attending surgeons, followed by a divisional multidisciplinary meeting which included all attending physicians, nursing, and house staffs at the end of the week. The coding of complications into the database from the previous week was reviewed and the nature of complications discussed.
