# Supplementary material for: Risk factors for prolonged air leak and need for intervention following lung resection
Source: Interact Cardiovasc Thorac Surg. 2021 Sep 18;34(2):212–8. doi: 10.1093/icvts/ivab243 (PMC8766207; doi:10.1093/icvts/ivab243)
Supplement: ivab243_Supplementary_Data [file ivab243_supplementary_data.zip › Supplemental Tables.docx]

Supplemental Table 1: Univariable and Multivariable logistic regression for overall complication*

|  | **Univariable** |  |  | **Multivariable** |  |  |
| --- | --- | --- | --- | --- | --- | --- |
| **Variable** | **Odds Ratio** | **95% CI** | **p-value** | **Odds Ratio** | **95% CI2** | **p-value** |
| Age 70 + (ref <70) | 2.41 | 1.95-3.00 | <0.001 | 2.5 | 2.00-3.14 | <0.001 |
| **Female Gender (ref male)** | 0.75 | 0.60-0.92 | 0.007 | 0.8 | 0.64-1.01 | 0.06 |
| **Resection Type** |  |  |  |  |  |  |
| **Wedge Resection** | Reference |  |  | Reference |  |  |
| Segmentectomy | 1.53 | 1.06-2.18 | 0.021 | 1.31 | 0.89-1.89 | 0.166 |
| lobectomy | 3.23 | 2.57-4.07 | <0.001 | 2.38 | 1.84-3.09 | <0.001 |
| **Method** |  |  |  |  |  |  |
| **Open** | Reference |  |  | Reference |  |  |
| Thoracoscopic | 0.28 | 0.21-0.36 | <0.001 | 0.43 | 0.31-0.58 | <0.001 |
| Robotic | 0.41 | 0.23-0.70 | 0.002 | 0.45 | 0.25-0.79 | 0.007 |
| Prolonged Air leak (ref none) | 4.59 | 3.19-6.61 | <0.001 | 3.26 | 2.21-4.80 | <0.001 |

*Includes any Clavian-Dindo Grade II-V complications excluding prolonged air-leak

Supplemental Table 2: Univariable and Multivariable logistic regression for overall complication in patients with prolonged air-leak*

|  | **Univariable** | | | **Multivariable** | | |
| --- | --- | --- | --- | --- | --- | --- |
| **Variable** | **Odds Ratio** | **95% Confidence Interval** | **p-value** | **Odds Ratio** | **95% Confidence Interval** | **p-value** |
| Age <70 years old (reference ≥ 70 years old) | 1.498 | 0.75-3.02 | 0.255 |  |  |  |
| Gender, female (reference male) | 1.281 | 0.64-2.59 | 0.487 |  |  |  |
| FEV1% <40% (reference ≥40%) | 2.423 | 0.61-11.92 | 0.226 |  |  |  |
| FVC equal of greater 110% (reference <110%) | 0.736 | 0.15-3.17 | 0.686 |  |  |  |
| DLCO% <50% (reference ≥50%) | 10  3.077 | 1.05-9.61 | 0.045 | 1.85 | 0.53-6.39 | 0.327 |
| Current smoker (reference quit >30 days prior) | 1.066 | 0.427-2.64 | 0.889 |  |  |  |
| Systemic steroid use, yes (reference no) | 3.648 | 1.17-13.79 | 0.035 |  |  |  |
| Ipsilateral radiation therapy, yes (reference no) | 0.741 | 0.24-2.19 | 0.592 |  |  |  |
| Neoadjuvant Chemotherapy, yes (reference no) | 0.811 | 0.29-2.16 | 0.677 |  |  |  |
| Prior ipsilateral Cardiothoracic surgery, yes (reference no) | 1.209 | 0.55-2.67 | 0.635 |  |  |  |
| Degree of resection (reference wedge) |  |  |  |  |  |  |
| Segmentectomy | 0.648 | 0.19-2.10 | 0.477 |  |  |  |
| Lobectomy | 1.157 | 0.52-2.60 | 0.721 |  |  |  |
| Method (ref thoracotomy) |  |  |  |  |  |  |
| Thoracoscopic | 1.161 | 0.53-2.59 | 0.711 |  |  |  |
| Robotic | 0.633 | 0.028-7.24 | 0.72 |  |  |  |
| Lysis of adhesions or re-do, yes (ref no) | 1.211 | 0.61-2.45 | 0.591 |  |  |  |
| Any interventions, yes (vs. no) | 4.106 | 1.71-10.72 | 0.002 | 4.39 | 1.04-23.45 | 0.056 |

DLCO= Diffusion Capacity for Carbon Monoxide; FEV1= Forced expiratory volume in 1 second; FVC= Forced Vital Capacity

*Includes any Clavian-Dindo Grade II-V complications excluding prolonged air-leak
